# Deformation and Fatigue Behaviour of A356-T7 Cast Aluminium Alloys Used in High Specific Power IC Engines

**DOI:** 10.3390/ma12183033

**Published:** 2019-09-18

**Authors:** Elanghovan Natesan, Stefan Eriksson, Johan Ahlström, Christer Persson

**Affiliations:** 1 Department of Industrial and Materials Science, Chalmers University of Technology, 412 96 Göteborg, Sweden; johan.ahlstrom@chalmers.se (J.A.); christer.persson@chalmers.se (C.P.); 2 Volvo Car Corporation, Analysis and Verification, 405 31 Göteborg, Sweden; stefan.a.eriksson@volvocars.com

**Keywords:** cylinder head, cast aluminium, A356, constitutive modelling, fatigue, plasticity

## Abstract

The continuous drive towards higher specific power and lower displacement engines in recent years place increasingly higher loads on the internal combustion engine materials. This necessitates a more robust collection of reliable material data for computational fatigue life prediction to develop reliable engines and reduce developmental costs. Monotonic tensile testing and cyclic stress and strain-controlled testing of A356-T7 + 0.5 wt.% Cu cast aluminium alloys have been performed. The uniaxial tests were performed on polished test bars extracted from highly loaded areas of cast cylinder heads. The monotonic deformation tests indicate that the material has an elastic-plastic monotonic response with plastic hardening. The strain controlled uniaxial low cycle fatigue tests were run at multiple load levels to capture the cyclic deformation behaviour and the corresponding fatigue lives. The equivalent stress-controlled fatigue tests were performed to study the influence of the loading mode on the cyclic deformation and fatigue lives. The two types of tests exhibit similar fatigue lives and stress-strain responses indicating minimal influence of the mode of loading in fatigue testing of A356 + T7 alloys. The material exhibits a non-linear deformation behaviour with a mixed isotropic and kinematic hardening behaviour that saturates after the initial few cycles. There exists significant scatter in the tested replicas for both monotonic and cyclic loading.

## 1. Introduction

Aluminium alloys are employed in the automotive industry because of their low density, high thermal conductivity and a good resistance to corrosion. The strength of aluminium could be increased by alloying with elements like copper, magnesium, silicon, manganese and zinc [1]. Al-Si alloy systems like A356 are commonly used for casting cylinder heads in internal combustion engines with complex geometries on account of their low melting point, high castability, lower shrinkage, good machinability, good corrosion resistance, high thermal conductivity and good mechanical properties [2,3,4]. The last decade has seen rapid improvements in the internal combustion engine (ICE) downsizing to reduce CO_2_ emissions while retaining the performance of previous generation engines through pressure charging [5,6]. This leads to its own accompanying problems with the chief of the concerns being the cylinder head durability on account of the increased thermal and mechanical loads [7]. Consequently, the cylinder head made of the A356 cast aluminium alloys are often employed in an overaged T7 condition on account of their increased thermal stability [8,9,10]. Further, A356-T7 is often added with some 0.5% copper to improve the elevated temperature strength at temperatures above 250 °C without a marked reduction in its ductility. The resulting microstructure and corresponding mechanical properties are often highly sensitive to the quench rate of the castings [11].

The cylinder head in an internal combustion engine experiences a wide variety of loads. The engine start-stop sequence and the non-uniform temperature distribution in a cylinder head often results in high temperature gradients and consequently, cyclic thermo-mechanical loads. The material is further subjected to mechanical loads arising from the camshaft and valve motions. Further, prior mechanical loads are placed during the assembly operations from bolts that connect the cylinder heads to the cylinder block and other accessories that are mounted to the structure [12]. Accordingly, the multiple failure modes act simultaneously on an engine cylinder head ranging from simple high cycle fatigue by induced elastic deformations to more complex thermo-mechanical fatigue failures that have severe associated material deformation and plasticity [11,12,13,14]. 

To predict the life of the component subjected to such loads, reliable constitutive models are needed to describe the cyclic stress-strain behaviour and fracture models to predict the life of the component from the load response history. In cases where substantial plastic deformation is observed, a strain-life approach based on Coffin-Manson like models are often employed to estimate the fatigue life, for instance, fatigue failures of the cylinder heads in load cases involving plasticity [14,15]. The strain-based approach can also efficiently handle load cases with mostly elastic deformation and, consequently, long lives such as in high cycle fatigue (HCF) and is thus, a comprehensive method to model fatigue lives [15]. The strain-based approach to predict the fatigue life employs the cyclic stress-strain curve to model the material response to various loads and the strain life Coffin-Manson type model to predict failure. It is often successfully employed in load cases involving high thermal stresses and strains associated with thermo-mechanical fatigue [15].

Koutiri et al. [16] extensively studied the fatigue behaviour of A356-T7 alloy and concluded that fatigue cracks mostly originated from micro-shrinkage pores and less frequently from other microstructural inhomogeneities. The study further established that there was no significant difference in the crack initiation or propagation between the multi-axial loading conditions and uniaxial loads with identical fatigue lives observed for both sets of loading. Fuoco et al. [9] studied the thermo-mechanical fatigue behaviour of custom cast cylinder heads and concluded along similar lines that micro porosity and oxide inclusions were the primary source of the low cycle fatigue crack initiation. Azadi et al. [17] studied the effect of heat treatment on A356 alloys and concluded that the heat treatment has a significant influence on the deformation and fatigue properties of the alloy, especially at lower temperatures with the differences getting lower with increasing testing temperatures. The study also compares the differences between strain and stress controlled monotonic deformation behaviour and perceives no significant difference in material response to the different loading modes. Takahashi et al. [18] studied the effect of over ageing of the peak aged A356-T6 material extracted from cylinder heads and observed that the thermal fatigue life improved with over-ageing, but with the effect on fatigue life improvement decreasing for increasing ageing times. Tabibian et al. [3,10] studied the isothermal low cycle fatigue (LCF) behaviour at 250 °C and out-of-phase thermo-mechanical fatigue (TMF) behaviour of unaged A356 and over-aged A356-T7 lost foam cast alloys and observed that the unaged A356 exhibited cyclic softening behaviour before subsequent stabilization whereas A356-T7 exhibited a relatively mild softening behaviour through its life. They also observed an insignificant difference in the cyclic deformation and fatigue behaviour of A356 alloys produced by lost foam casting and die casting methods. The study also demonstrated how plastic dissipated energy per cycle could be effectively used to assess TMF life of die-cast aluminium alloys using model parameters obtained using isothermal LCF tests. Barlas et al. [12] studied the influence of over-ageing on the thermo-mechanical fatigue life of cylinder heads made of A356-T7 alloys and concluded that the ageing of the material as a function of time and temperature needs to be considered together with low cycle fatigue isothermal plasticity at various temperatures for accurate non-isothermal computational modelling of fatigue lives associated with the TMF load cycles. Bingrong et al. [19] studied the effect of the inter dendritic arm spacing (DAS) on the mechanical properties of A356-T6 alloys and observed that the ultimate tensile strength and elongation were strongly affected by the DAS while the yield strength exhibited a weak dependence on the DAS. But, the influence of ageing had a more profound effect on the yield strength than the ultimate tensile strength and ductility.

While there are numerous studies documenting the deformation and fatigue properties of peak aged (T6) A356 alloys [18,19,20], there is a dearth of relevant literature studying the deformation and fatigue behaviour of the overaged (T7) A356 group of alloys with copper additions. Since numerous studies have showed the importance of considering the heat treatment effect on the mechanical deformation and fatigue behaviour [3,17,18], this study aims to bridge the knowledge gap on the deformation and fatigue behaviour of the over-aged A356 + 0.5 wt.% Cu-T7 group of alloys. The cast structures often have residual stresses from the quench process associated with the heat treatment and in addition are likely to have mechanical assembly loads [11]. Since the operational loads are further superimposed on the prior loads, it is of interest to study the cyclic deformation behaviour of the material when subjected to asymmetrical loads. It is also of interest, considering the different loading modes of the cylinder head, to study the equivalence of stress and strain-controlled fatigue tests for A356-T7 alloys. While such a study exists for steels [21], no such research work has been published for cast aluminium alloys. 

To encapsulate the aims and methods of the paper, monotonic testing is performed to capture the uniaxial stress-strain behaviour, completely reversed cyclic strain-controlled testing is conducted at different load levels to capture the continuum deformation behaviour and the fatigue lives of the material. The cyclic strain-controlled tests are also run with tensile and compressive mean strains to study the mean stress relaxation and the effect of such loads on fatigue lives. To compare the difference between the loading modes on the deformation and fatigue behaviour, equivalent cyclic stress-controlled fatigue tests are run with the stabilized stress values obtained from the corresponding strain-controlled tests. The obtained monotonic, cyclic hardening curves are then modelled using a Ramberg-Osgood type model and the continuum deformation behaviour is modelled using a non-linear kinematic and isotropic combined hardening model.

To have useful numerical models that accurately capture the deformation and fatigue behaviour of the A356-T7 material at critically loaded areas of a cylinder head and to eliminate material variability, the test specimens are extracted only from the highly loaded valve bridge areas of Volvo Cars’ inline 4 cylinder VEP4 petrol engines. The study comes with the limitation of considering only a constant set of processing parameters and microstructural conditions for this study on deformation behaviour, life and numerical modelling. Attention should be paid to the fact that the manufacturing processes have a significant influence on the nature of the castings and the resulting microstructure, even for identical chemical compositions, which tends to have a dramatic effect on both the deformation and fatigue lives. The obtained constitutive and fatigue life models could be used to replicate the complete behaviour of the said material in component design and life estimation.

## 2. Materials and Methods 

### 2.1. Material

**Figure 1:**
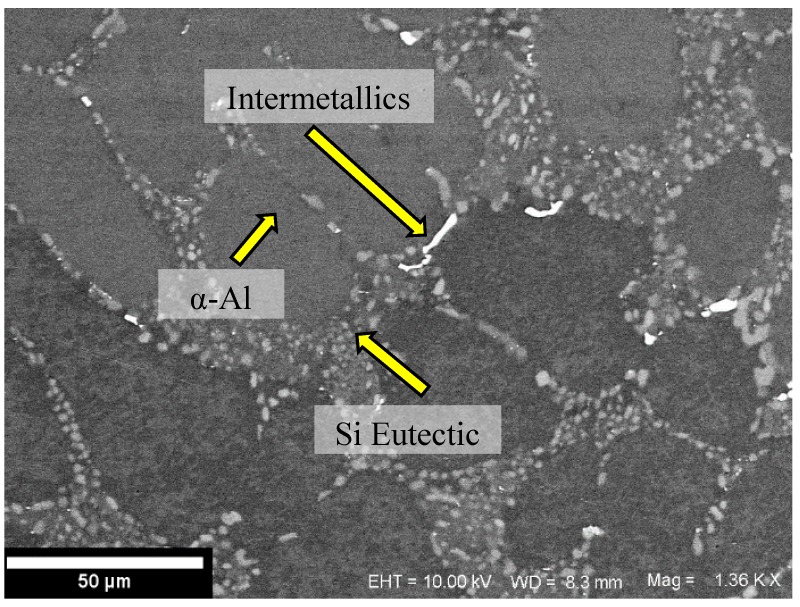
Electron micrograph illustrating the phases present in the tested cast A356-T7 + 0.5% Cu alloy.

The average chemical composition of the alloy based on two measurements is presented in Table 1. A representative microstructure of the alloy from the cylinder head is shown in Figure 1. A Zeiss LEO 1550 SEM (Zeiss, Jena, Germany) was used to obtain the microscopic image presented in Figure 1. A Gemini field emission gun (Zeiss, Jena, Germany) was used with an acceleration voltage of 10 kV to minimize the influence of the underlying matrix material. The software Aztec 3.1 (Oxford Instruments, Abingdon, England) was used to process the electron micrograph. The secondary dendrite arm spacing (SDAS) measured using the mean linear intercept method on the aligned sets of secondary cells was between 30–32 µm in the studied region of interest. 

The dendritic structure of the cast alloy can clearly be seen with the eutectics flanked by primary aluminium. The A356-T7 samples tested can broadly be said to have three different components in the microstructure as indicated in Figure 1: 

Primary α-Aluminium solid solution phase, the Al-Si eutectic and other intermetallics that encompasses intermetallic compounds from excess Mg, Cu, Fe and Mn not in the solid solution of the α-Aluminium phase. 

The size, volume, morphology, time of formation etc., of the said intermetallics depend on the specific alloy composition, solidification and heat treatment conditions and together have a profound impact on the deformation characteristics of the material [22].

### 2.2. Material Extraction for Testing

The samples for testing have been extracted from Volvo Cars’ (Gothenburg, Sweden) inline 4 cylinder VEP4 petrol engine cylinder heads. The cylinder heads were manufactured by the gravity tilt die casting method with the molten alloy temperatures maintained between 690–710 °C. The die temperatures were maintained between 200–240 °C with the fire deck area having a water-cooled side to promote faster directional solidification. Nitrogen was used to degass the component with the aid of graphite rotors. Titanium was used for grain size refinement while strontium additions were employed for eutectic modification. The T7 over-ageing was performed by solutionizing the cast component for approximately 3 h at temperatures above 530 °C followed by air cooling and a final artificial ageing at temperatures between 200–240 °C for up to 5 h. 

**Figure 2:**
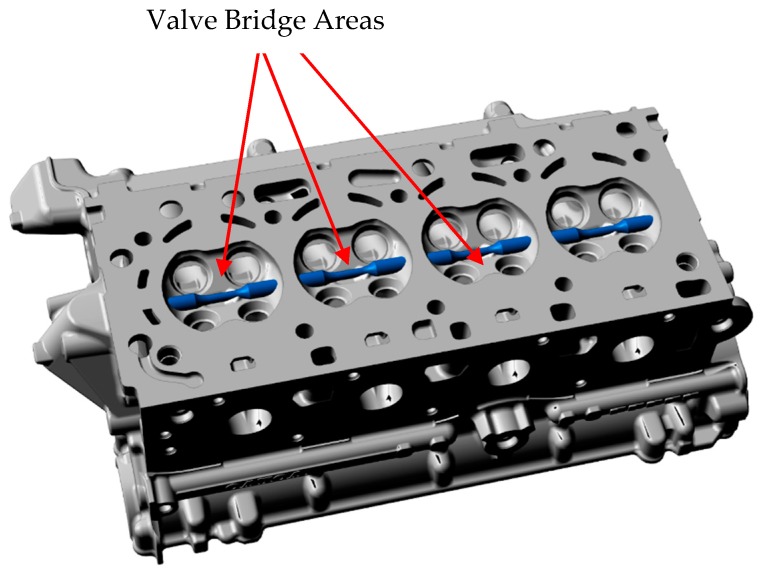
Specimen extraction locations from the cylinder heads.

**Figure 3:**
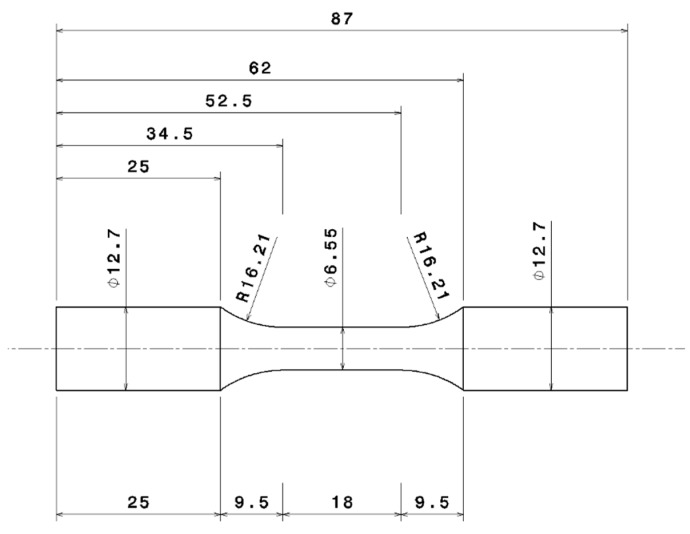
Specimen geometry machined conferring to the ASTM standards.

For this study, the test specimens were extracted from regions with close proximity to the highly loaded areas in the cylinder heads. The valve bridge area as indicated by the arrows in Figure 2 in the fire deck is highly susceptible to damage under TMF loadings [8,14,18,23] and hence, is the area of interest in this study. The material closest to the surface is extracted and machined to the geometry as shown in Figure 3 in accordance with the ASTM standards [24,25,26]. The uniaxial loads are applied to such specimens with a long straight cylindrical gauge area with the extensometer mounted.

### 2.3. Testing

The mechanical tests were performed with a uniaxial Instron 8501 servo-hydraulic testing machine (Instron, Norwood, MA, USA) and a 1 kHz high frequency data acquisition system was employed to record the load-response data. The strains in the specimens were measured by clipping on a Instron 2620-603 axial dynamic extensometer (Instron, Norwood, MA, USA) mounted on the gauge length [27]. 

Three identical monotonic tensile tests were performed using strain control and with a constant strain rate of 10^−4^ s^−1^ in accordance with the ASTM standard for tensile tests [24]. The samples were strained until fracture to estimate the ductility and toughness of the alloy. 

The strain-controlled fatigue tests were performed with three different total strain amplitudes of 0.2, 0.3 and 0.4% and a completely reversed strain ratio of *R* = −1 with three replicas at each level. A constant strain rate of 1% s^−1^ was used for the strain cycling that which was indirectly set by using the corresponding constant loading frequencies while employing a triangular wave form of the load cycles. To study the effect of the mean strain on the corresponding stress evolution, strain-controlled tests were carried out with the mean strains of +0.2% and −0.2% with a strain amplitude of 0.4%. In both cases, the tests were run at the same constant strain rate of 1% s^−1^ identical to the prior completely reversed strain-controlled tests. All the tests were run to failure to obtain the strain-life curves for the material. The fatigue tests were aimed at developing a wide-ranging strain-life curve that could even be used in situations where there is little plastic deformation and long lives are anticipated. Under such low load conditions (for e.g., fatigue tests at total strain amplitude of 0.2%), the surface irregularities could significantly influence the measured life of the test specimens [15]. Therefore, the test samples were ground and polished to a mirror surface finish. 

For the stress-controlled tests, the peak stresses recorded at half the life of one of the strain-controlled tests (*N*_f_/2) were used as the basis. The samples were cycled between the minimum and maximum stresses obtained at the stabilized stage of the equivalent strain-controlled tests. The tests were run at the same frequency as the equivalent strain-controlled tests and with triangular load wave forms. There is a small deviation from the constant strain rate of 1% s^−1^ used in the equivalent strain-controlled tests, but their effect on the resulting mechanical properties are expected to be negligible for the face centred cubic (FCC) metals.

To estimate the fatigue life of the tested samples in strain-controlled fatigue tests, a failure is assumed to have occurred when the peak stress recorded during a loading cycle drops significantly (below 80%) compared to a reference stabilized hysteresis loop, taken as the 25th strain cycle in this case. For the stress-controlled tests however, the load cycles between any significant change of strain amplitudes and macroscopic fractures were negligible, i.e. the number of cycles to rupture equate to the life of the specimens *N*_f_. A brief summary of the complete test plan is as presented in Table 2 below.

## 3. Results

### 3.1. Monotonic Deformation

**Figure 4:**
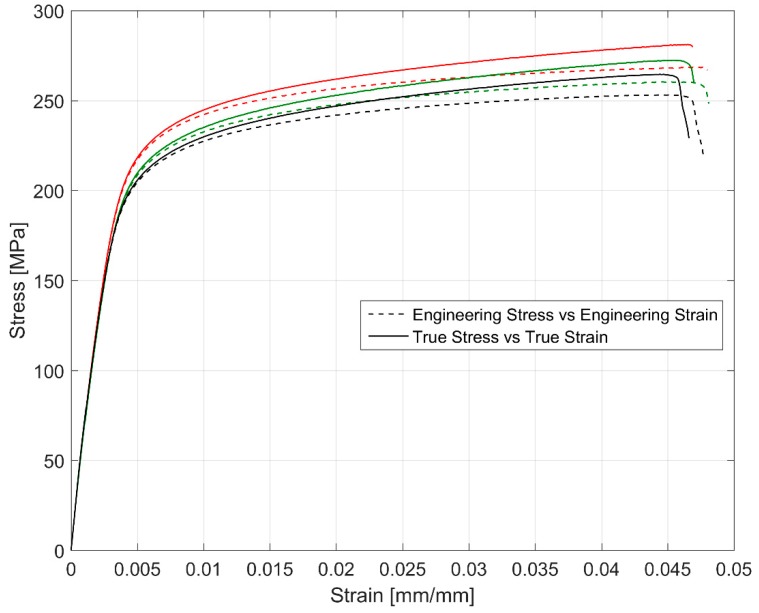
Monotonic tensile test results of 3 tested specimens.

Figure 4 shows the stress development for strain controlled monotonic deformations of three specimens at a strain rate of 10^−4^ s^−1^. Since the true strain and true stress differ considerably from engineering measures at higher deformation levels, Figure 4 shows both the true stress versus true strain and the corresponding engineering stress versus engineering strain values. Considerable difference in the deformation behaviour can be seen in the tensile curves once significant macroscopic plasticity sets in. All the samples exhibit strain hardening post yield with comparable fracture strains. While all the different specimens show a gradual change from elastic deformation to plastic deformation without a distinct yield point, there exists about a 6% variation in the yield and ultimate tensile strength estimated for the tested samples with reference to the strongest tested sample. As detailed earlier, it is to be expected considering the inter dendritic arm spacing variation within the sample extraction zones and the strong correlation of the inter dendritic arm spacing to the mechanical properties of A356-T7 group of alloys [28]. Table 3 summarizes the estimated monotonic tensile properties of the three tested samples. 

### 3.2. Strain Controlled Fatigue Tests

#### 3.2.1. Stress Evolution

**Figure 5:**
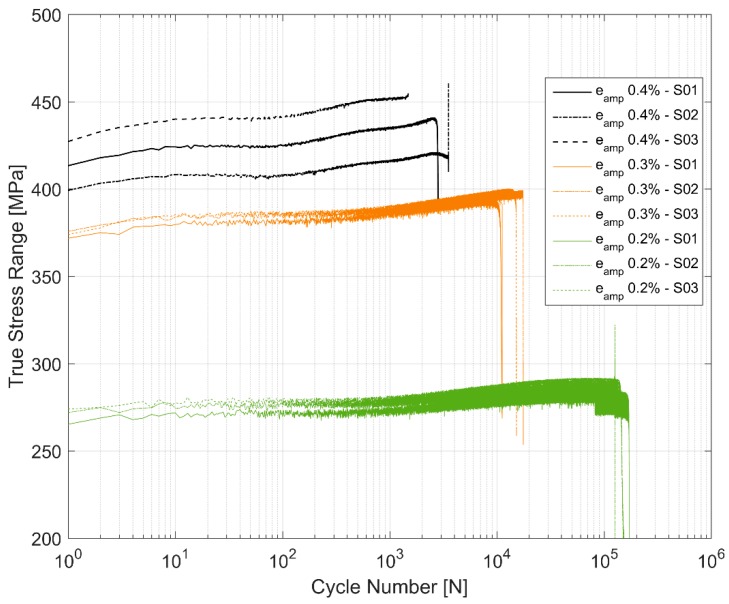
True stress range development.

**Figure 6:**
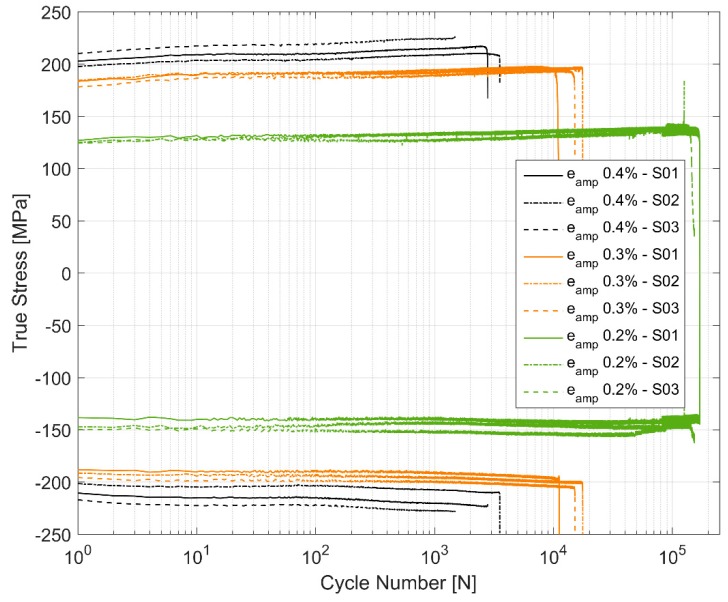
True stress peaks under tension and compression developed for each strain cycle.

Figure 5 shows the true stress range development through the life for the three samples tested at each of the three strain amplitude levels. The stress range increases with successive cycling of the prescribed strains indicating a cyclic hardening behaviour of the material. While the material hardens cyclically, it does so with some asymmetry between the peak tensile and compressive stresses as shown in Figure 6. The material seems to exhibit a probable Bauschinger like effect [29] causing a higher peak compressive stress development for all the cases as all the tests were started with loading in tension followed by load reversal to compression. This evidences a material dependent deformation behaviour. Furthermore, of interest to note is the variation in peak stresses and their development especially at the higher cyclic load level of 0.4% as shown in Figure 5. The life of the component at 0.4% amplitude strain cycling seems to be strongly related to the peak tensile stress response that the material develops with the life of the component decreasing with higher peak tensile stress development. The other two tested amplitudes of 0.2% and 0.3% not only show no such trend, but also show significantly less scatter in stress development between the different test samples. The hardening of the material through the life varies between approximately 7–9% as summarized in Table 4.

#### 3.2.2. Plastic Strain Evolution

**Figure 7:**
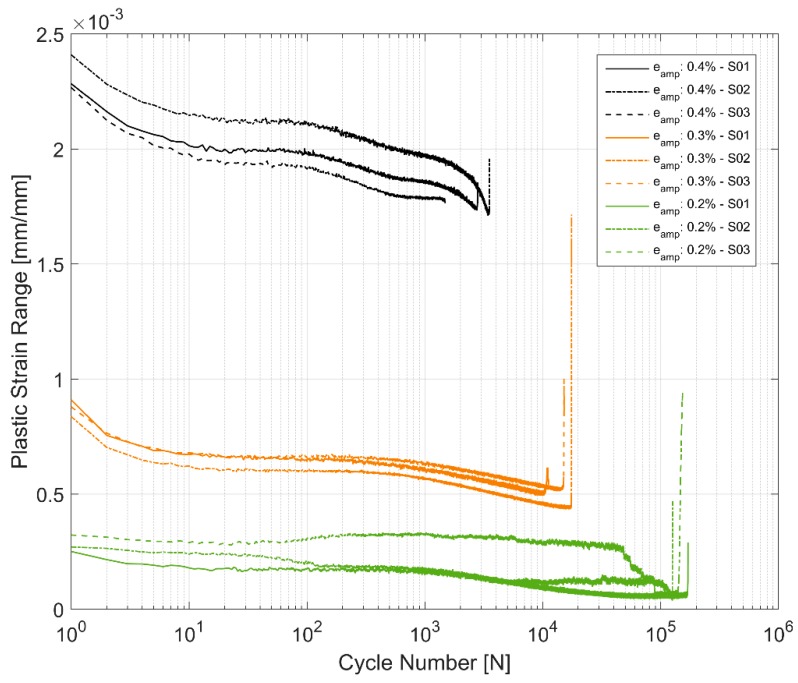
Plastic strain range evolution.

Another way to look at the influence of the cyclic load on the hardening behaviour is to look at how the plastic strain develops with the cyclic strain loading. As can be seen in Figure 7, the plastic strain range keeps decreasing with successive strain load cycles for all strain levels and specimens. The plastic strains measured at 0.2% strain load is very low and cannot be meaningfully used to generalise material behaviour and will henceforth not be considered for further analysis or discussions. However, the plastic strain range shows similar trend for tests at 0.3% and 0.4% strain amplitude cycling. The material demonstrates an initial steeper hardening curve before levelling off after approximately 30 cycles before showing an increased hardening rate closer to failure. The reduction in the width of the hysteresis loop is approximately 20% at higher test strain amplitudes and increases to approximately 40% at 0.3% strain amplitude. This contrasts with the stress response where the hardening stays relatively the same at approximately 8% as described before in Table 4. 

#### 3.2.3. Cyclic Yield Evolution

**Figure 8:**
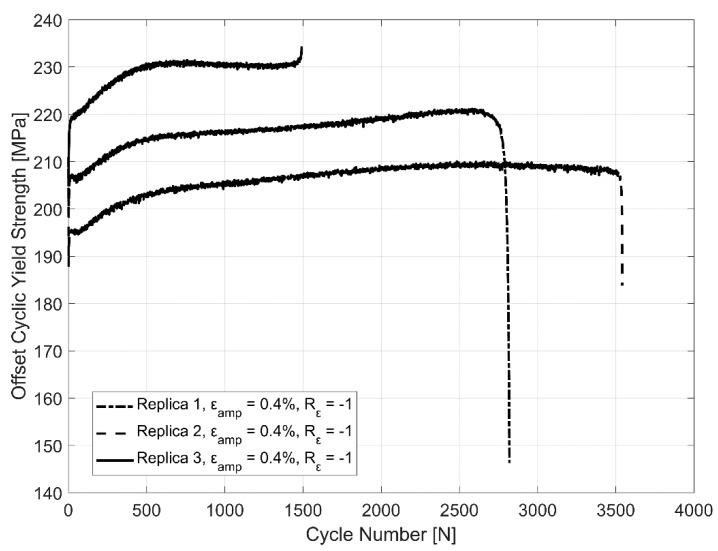
The evolution of offset yield strength in cyclic loading at ε_amp_: 0.4% of 3 identical replicas.

The constant reduction of the plastic strain amplitude implies a constantly hardening behaviour and the corresponding behaviour is reflected in the evolution of the tensile off-set yield strength (0.2%) estimated in cyclic loading between the identical replicas tested between the total strain levels of ±0.4% as shown in Figure 8.

#### 3.2.4. Effect of Mean Strain

**Figure 9:**
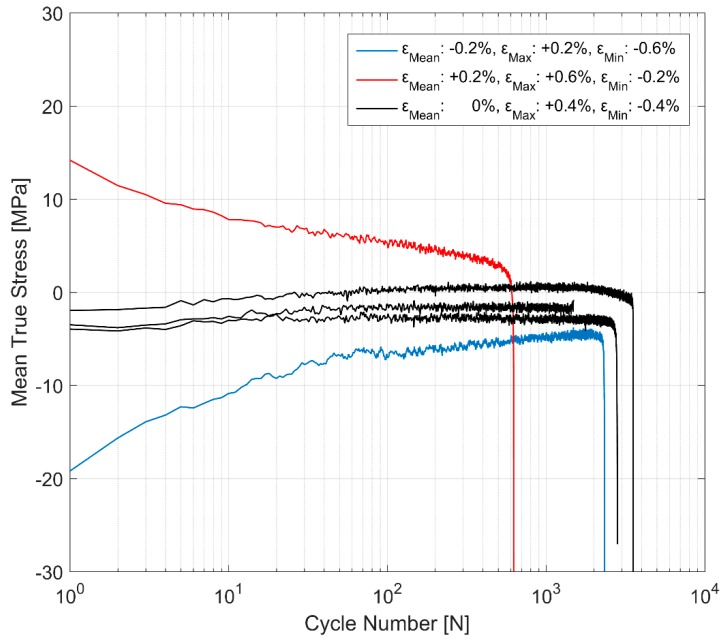
The effect of mean cyclic strains on mean stress evolution.

**Figure 10:**
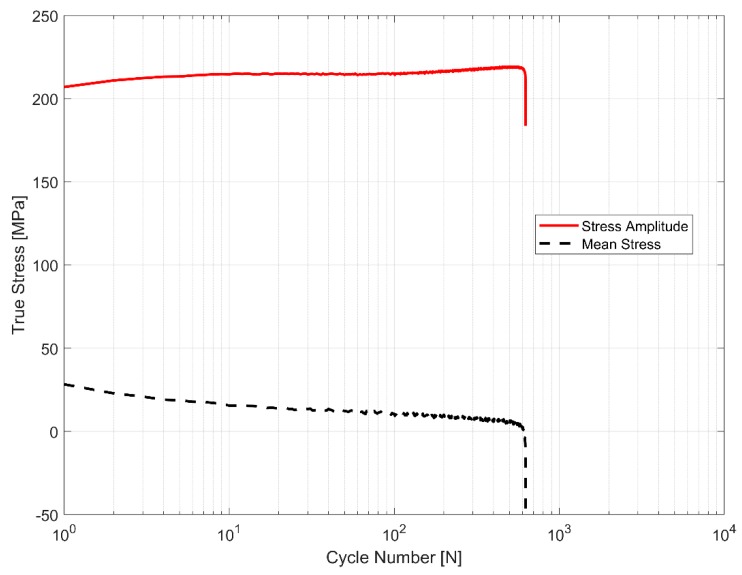
The interplay between cyclic hardening and mean stress relaxation with a tensile mean stress.

**Figure 11:**
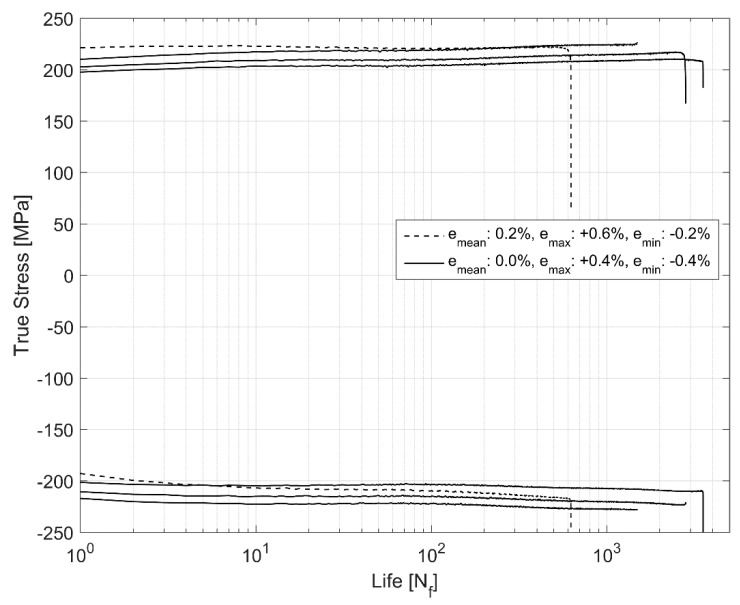
True stress evolution of symmetric and biased strain limits in cyclic loading.

The initial strains are often observed in the cylinder heads of internal combustion engines on account of the assembly and operational engine loads [17]. Hence, it becomes imperative to explore the effect of more complex non-completely reversed cyclic strain loads (R_ε_ ≠ −1) on the cyclic deformation behaviour of the A356-T7 material. To study the effect of such loads on the deformation and fatigue behaviour of the material, two strain load cycles, one with the total strain values between ε_max_ = 0.6% and ε_min_ = −0.2% and the other within the total strain limits specified between ε_max_ = 0.2% and ε_min_ = −0.6% were tested and the resulting mean stress development is presented in Figure 9. The material exhibits cycle-dependent mean stress relaxation. A stabilized mean stress is not achieved during the life of the specimen in the strain controlled cyclic loading and the material exhibits continuous non-linear mean stress relaxation with a strong initial relaxation that reduces gradually as the cyclic loading progresses through the life of the specimen. Figure 9 and Figure 10 show the interaction between the simultaneous hardening and the mean stress relaxation in the cyclic strain-controlled test with tensile mean stress. While the material does harden, the cycle dependent mean stress relaxation seems to be more strongly influenced by the increase in compressive peak stresses as can be seen in Figure 11. 

### 3.3. Stress Controlled Fatigue Tests

To study the equivalence of stress and strain-controlled tests on the stress-strain response and the fatigue lives, the peak stresses developed during the strain-controlled tests were replicated through force-controlled loading to study the corresponding strain evolution. The stress response obtained with the asymmetric strain loading exhibiting the mean stress relaxation was replicated in the stress-controlled tests as well. The stress amplitudes obtained in the strain-controlled tests that were used in the stress controlled cyclic loading tests are presented in Table 5.

As can be seen in Table 5, three of the tests have a compressive mean stress corresponding to the stable response taken at N_f_/2 of the strain controlled tests with a zero main strain. The last stress controlled test corresponds to the stable stress-strain behaviour taken at half the life of the strain controlled test with a 0.2% mean strain and a strain amplitude of 0.4% as described in Section 3.2.4 Effect of Mean Strain.

#### Strain Evolution

**Figure 12:**
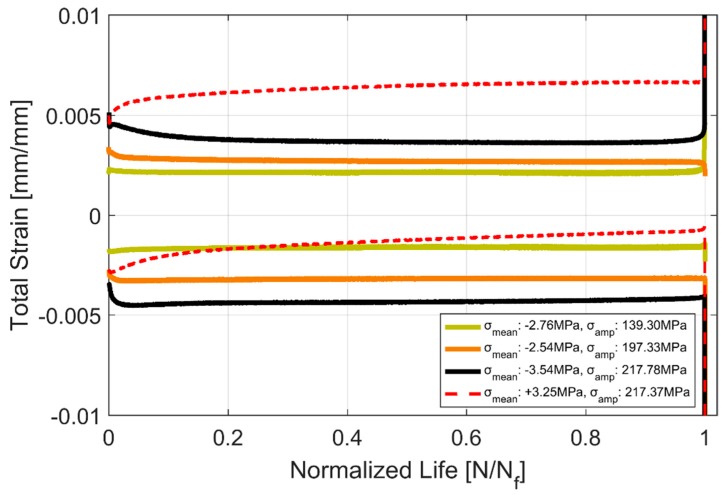
Strain evolution in equivalent stress controlled cyclic tests.

**Figure 13:**
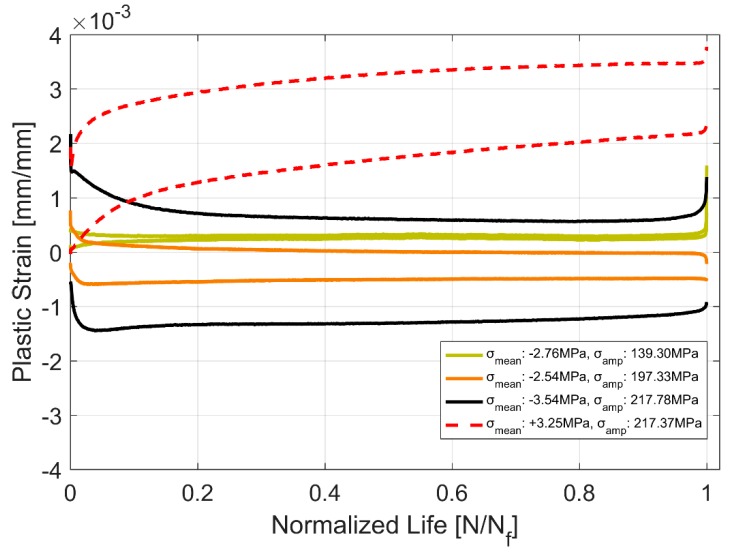
Plastic strain evolution in equivalent stress controlled cyclic tests.

As with most metals that exhibit mean-stress or cycle-dependent relaxation in strain controlled cyclic loading with a positive mean strain, A356-T7 also exhibits the phenomenologically complimentary cycle-dependent creep, that is also otherwise known as ratchetting, with a positive mean stress [15] as can be seen in Figure 12 and Figure 13. The stress controlled cyclic loading with compressive mean stresses result in a strain response that is fairly stable after the initial few cycles, whereas the cyclic loading with a tensile mean stress exhibits the ratcheting behaviour up until failure. All the tests exhibit a stronger initial rate of hardening that stabilizes to varying degrees over the course of the life of the component.

In stress controlled cyclic loading with the positive mean stress, the plastic strain accumulates as the material cyclically accumulates plastic strain, i.e., ratchets with successive loading cycles and eventually accumulates and fails similar to tensile tests [15]. A comparison of the fatigue lives of equivalent stress and strain controlled specimens is presented in Table 6 below.

**Figure 14:**
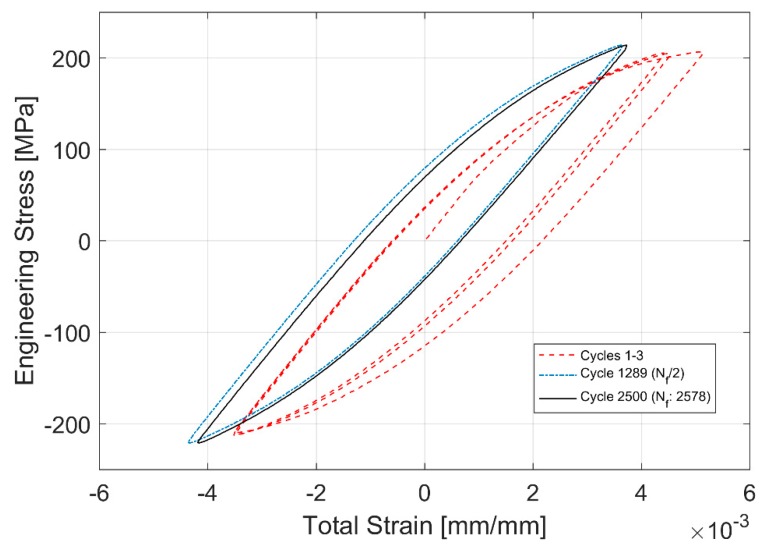
Hysteresis Loop development through life for stress controlled fatigue tests with a compressive mean stress of −3.54 MPa.

**Figure 15:**
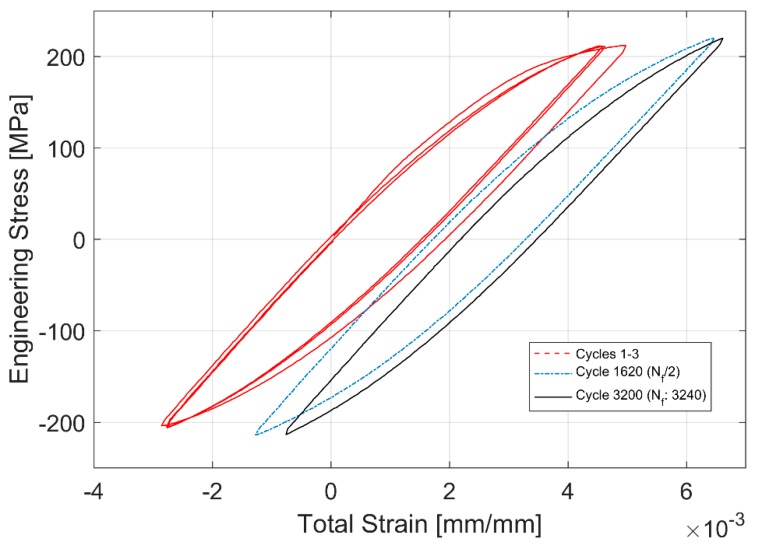
Hysteresis Loop development through life for stress controlled fatigue tests with a tensile mean stress of +3.25 MPa.

The evolution of the hysteresis loops during the stress controlled fatigue tests is presented in Figure 14 and Figure 15. The stress controlled fatigue tests with the compressive mean strain of −3.54 MPa and a stress amplitude of 217.78 MPa are presented in Figure 14 and the corresponding equivalent stress controlled tests with the tensile mean stress of +3.25 MPa and a stress amplitude of 217.37 MPa are presented in Figure 15. The specimen with the tensile mean strain exhibits significant ratcheting up until failure compared to the test with a compressive mean strain where stabilization is reached fairly quickly through the life of the specimen. This can be attributed to the changes in area where the true stress increases in tension and decreases in compression. Since the test control is based on the engineering stress values, it is quite common to observe this accumulation of plastic strains and ratcheting in stress-controlled fatigue tests with a tensile mean stress.

### 3.4. Comparison of Monotonic and Cyclic Hardening Curves

**Figure 16:**
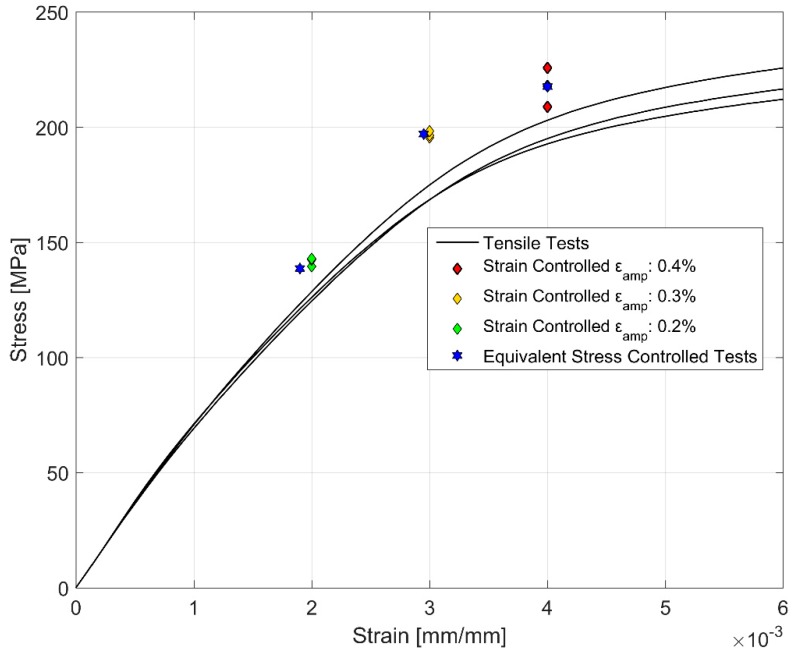
Comparison of monotonic vs cyclic hardening.

Figure 16 compares the monotonic deformation curves against the stress and strain amplitudes obtained from the strain and stress-controlled fatigue tests. The stress and strain amplitudes for the cyclic deformation curves were obtained at half the life of the corresponding specimens representing the stable cyclic deformation behaviour [15]. It can clearly be observed from Figure 16 that A356-T7 hardens cyclically as depicted by the higher stress response developed in cyclic loading at comparable strains relative to the monotonic deformation.

## 4. Discussions

General Discussions:

The samples for testing were extracted from cast cylinder heads made of A356-T7 cast aluminium alloy. While the specimens could also be made in special moulds with the desired specimen geometry, such samples will often not have the same microstructural characteristics of industrially produced cylinder heads with complex geometries [4,16]. Hence, the study was performed with the samples extracted from critically loaded sections of full-scale industrially produced cylinder heads. 

The mechanical properties of the A356-T7 cast material depends highly on the cooling rate employed during solidification [19,28]. The studies by Carrera et al. [30] showed that the microstructural refining translated to the inter dendritic arm spacing was the single most dominant microstructural characteristic determining the deformation behaviour of A356 alloys and how the dendritic arm spacing and the size of the other constituents could be controlled by the cooling rate employed during the casting process. Since a complex cast structure like a cylinder head has a wide range of solidification microstructures at different regions, a wide spectrum of the alloy’s mechanical properties is found, depending on where the test specimens are extracted [19,30,31]. Further, owing to the varying thickness and geometry in the cylinder head, one can reasonably expect an uneven distribution of voids, impurities, shrinkage pores and cracks within the structure [13]. As to the microstructure observed, the interconnected fibrous phase distribution in the eutectic structure of the extracted samples comes from the potential sodium modification [4,32] that gives better toughness and ductility with the sodium content measured at 0.0014% from the chemical analysis. Boron and titanium are often added to cast aluminium to refine the grain size of the cast structure by promoting the nucleation rate during solidification [4,30].

While rotating beam fatigue tests are frequently used for determining the fatigue life curves of cylinder heads made of cast iron, the uniaxial tests are usually preferred for non-ferrous alloys [13] and hence used for the A356-T7 samples. Despite the enormous efforts to maintain the homogeneity and uniformity of the specimens used for testing, the observed scatter in the different tested mechanical properties are to be expected. All the variables in addition to the differing geometries and proximity to the mould walls influence the local cooling rate and the associated variation in the inter dendritic arm spacing (both primary and secondary) [28] affect the microstructure resulting in non-uniform mechanical properties as evidenced by the scatter in for example Figure 4 and Figure 5. 

### 4.1. Monotonic Deformation

**Figure 17:**
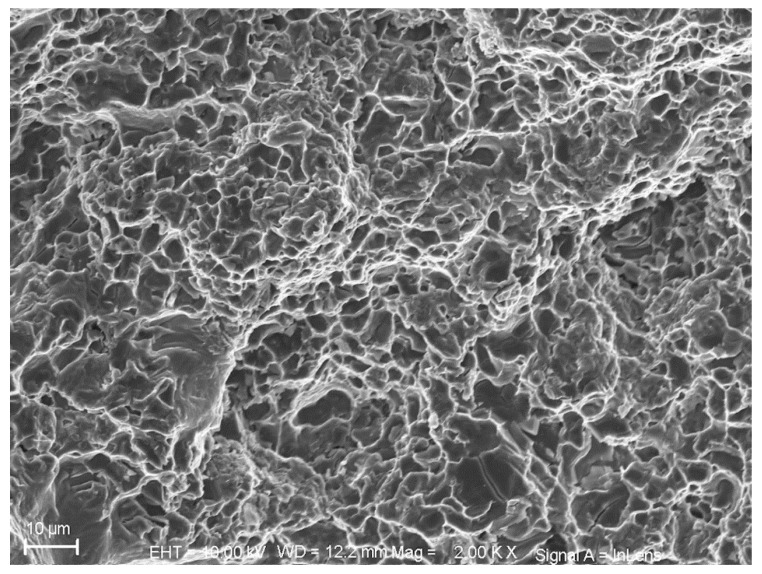
Monotonic fracture surface showing cup and cone or dimple features characteristic of a ductile fracture.

The tensile tests were performed at a constant total strain rate of 1.10^−4^ s^−1^. The monotonic tensile response of the material could be affected by the loading rate if viscous effects are involved, but for the fcc materials tested at room temperature as the A356 alloy, such effects are often negligible [15]. Regarding the tensile deformation behaviour of the A356 alloys, the non-linear hardening behaviour of the material could perhaps be as explained by Caceres et al. [22] who theorized that the Si, Cu, Mg and Fe rich intermetallics secondary particles performed akin to the reinforcement particles in a typical metal matrix composite while investigating the uniaxial tensile deformation behaviour of the Al-Si-Cu-Mg alloys similar to the A356 tested in this study. As the material is deformed, the hard intermetallic particles continue deforming elastically as the neighbouring material begins flowing plastically. High stresses are developed in the elastically deforming intermetallic particles resulting in significant hardening in the initial stages of the plastic deformation. As the applied total strain grows beyond 1–2%, there is a reduction in the tensile hardening rate as plastic relaxation is observed around the elastically deforming intermetallics and secondary phases, thus resulting in the characteristic non-linear uniaxial tensile deformation curve. Figure 17 below shows the fracture surface of the material after monotonic testing exhibiting the characteristic ductile fracture features.

The monotonic tests of A356-T6 documented by [17,33], show a higher yield and tensile strength in the range of 270 MPa and 330 MPa respectively on account of the material being peak aged. With the subsequent over ageing of the alloys, the coarsening of the secondary intermetallic precipitates is often observed, thus resulting in less hindrance to the movement of the plasticity inducing dislocation motion and hence, a reduced strength of the overaged A356 alloy under monotonic loading [18].

The cracks during a tensile fracture are often found to originate from hard silicon eutectic particles and the secondary intermetallic precipitates. These cracks coalesce with increasing deformation ultimately resulting in a tensile fracture as shown by Wang [33] and corroborated by [22,34]. The deformation studies by Zhu et al. [34] on A356-T6 alloys without copper show a tensile strength in the range of 150–179 MPa and UTS in the range of 250–285 MPa with higher ductility (5–10%) exhibiting a lower yield strength, but higher tensile strength and elongation in relation to the A356 + 0.5 wt.% Cu-T7 that was tested in this study. While A356 could indicate a wide latitude of chemical compositions, a rule of thumb to translate the tensile test results within the latitude of compositions often ascribed to A356 alloys could be as summarized by Caceres et al. [22]:

- The yield strength of the material often increases with increasing magnesium or copper content, but comes at the cost of lowering the ductility of the material.
- Iron is detrimental to both the strength and ductility of the material.
- With respect to microstructures, the dendrite arm spacing that was a consequence of the local cooling rates had a profound influence on the ultimate strength and the ductility of the alloy. This often lowered the ductility and ultimate tensile strength with increasing dendritic arm spacing (or a decreasing local solidification rate), while the flow stress and the strain hardening rate were faintly affected [22].

### 4.2. Strain Controlled Fatigue Tests

As with most metals, the tested samples of A356-T7 exhibit a nonlinear hardening behaviour with significant hardening in the initial cycles and the hardening rate decreasing for the successive load cycles through the life of the test specimen [15]. Figure 7 shows a continuous, but non-linear reduction in the width of the hysteresis loops as the plastic strain range reduces with successive load cycles. 

This non-linear hardening behaviour through the life of the tested A356 samples is similar to the one observed by Hauenstein et al. [35] while studying the cyclic straining of Al-2.8% Cu. That study investigates the role of two types of dislocation structures leading to the non-linear cyclic hardening behaviour of the alloy, namely the dislocation lines and dislocation tangles. The piling up of these dislocations at and in between the intermetallics and secondary phase precipitates obstruct the motion of such dislocations leading to hardening. While the hardening associated with the increase in the density of the dislocation tangles proceeded until the sample failed, the cyclic hardening associated with the dislocation lines were only present for a portion of the life of the component. The density of the said dislocation lines increase for a certain number of cyclic strain loadings with corresponding increase in hardening and a balance is reached between the formation of new dislocations and the annihilation of existing ones after a certain number of load cycles. The dislocation lines from the multiple slip planes present also adopt a more regular structure through the strain cycle loads and appear as highly resolved dislocation networks with increasing load cycles. The evolution of yield strength with successive strain load cycles as presented in Figure 8 indicates that the hardening rate is higher until approximately the 500th cycle with much less cyclic hardening exhibited in subsequent load cycles, indicating the probable role of such dislocation structures in the hardening behaviour as described above.

**Figure 18:**
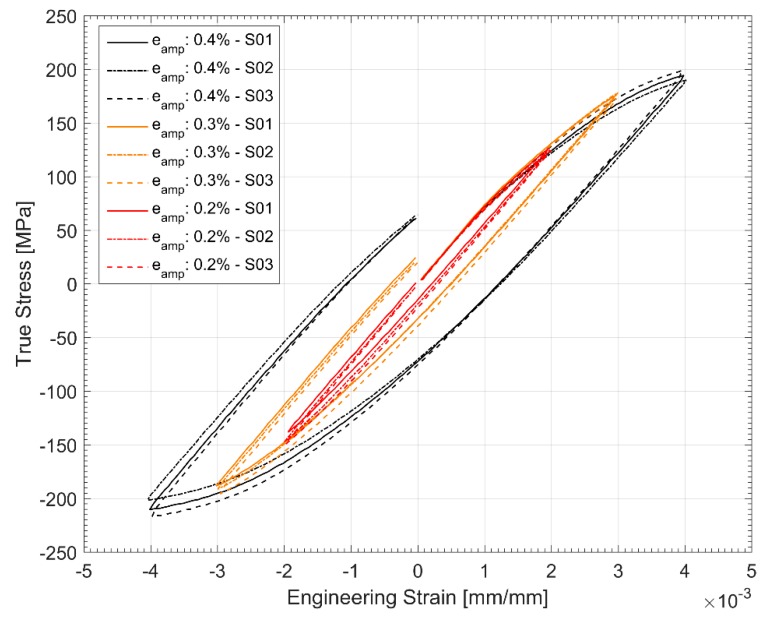
First cycle hysteresis loops of strain-controlled tests.

**Figure 19:**
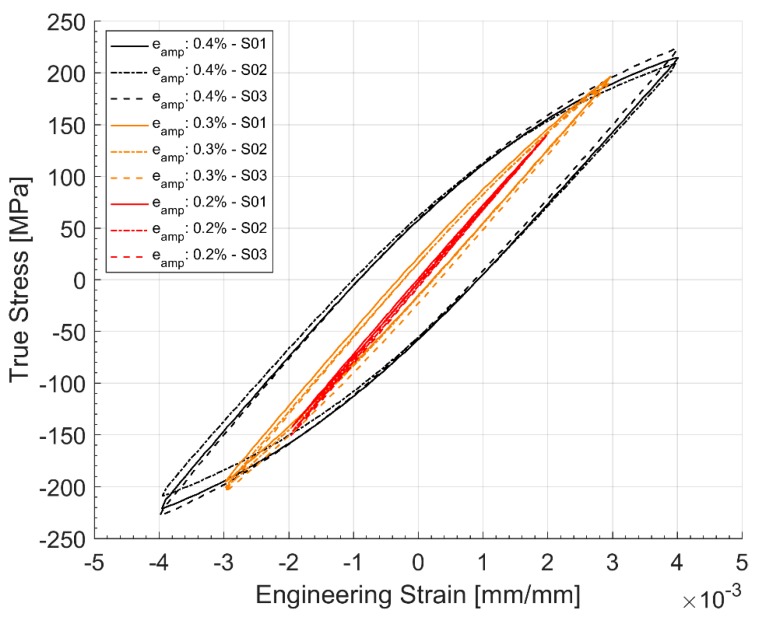
Half-life (Nf/2) cycle hysteresis loops of strain-controlled tests.

Figure 18 and Figure 19 show the hardening behaviour evolution of the A356 + 0.5 wt.% Cu-T7 alloys. As can be seen in the hysteresis loops, the plastic strains have reduced significantly for all load levels and is almost fully elastic for strain cycling between the total strain levels of ±0.2% at the half life. Furthermore, the differing hardening slopes between the strain levels at half-life compared to the first cycle hysteresis loops where the initial hardening slopes are similar. Similar observations have been made by Snowden [36] while studying the structures of dislocations in aluminium alloys during cyclic loading. Snowden contends that the differing hardening behaviour with differing load levels could be attributed to the enhanced reversibility, i.e., the Bauschinger strain, of the dislocations at lower strain loads under cyclic loading. In other words, Snowden observed that with reducing cyclic strain loads, the ratio of the Bauschinger strain to the total applied strain amplitude increased causing the disparity in the hardening curves at the different applied load cycles. 

Similarities could also be drawn with the studies by Grosskreutz [37] regarding the difference between monotonic and cyclic hardening curves. Grosskreutz observed a differing dislocation cell structure depending on the nature of the applied strain load being monotonic or cyclic. Regardless, smaller cell structures were associated with higher flow stresses in both cases. To explain the differing hardening levels with different strain load levels, Grosskreutz explains that at lower strain loads, the concentration of the dislocation structures increased with the increasing plastic strain levels and such structures were often observed predominantly in the primary slip planes. A fragmentation of such dislocation cell structures is often observed at increased applied strain amplitudes with the density of the dislocations now increasing in the secondary slip planes and forming a three-dimensional dislocation cell structure. In the study, it was also observed that the density of the point defects increases with increasing load cycles resulting in higher drag stresses on dislocation motions resulting in a non-linear hardening behaviour. Similar dissimilarities between the dislocation structures under monotonic and cyclic loading was also noted by Snowden [36] whose TEM observations exhibited dislocation tangles and loops under cyclic loading while more defined dislocation cell structures were observed under monotonic loading. A similar difference in the dislocation structures could explain the differing hardening behaviour observed between the monotonically and cyclically deformed A356-T7 samples as presented in Figure 16. 

**Figure 20:**
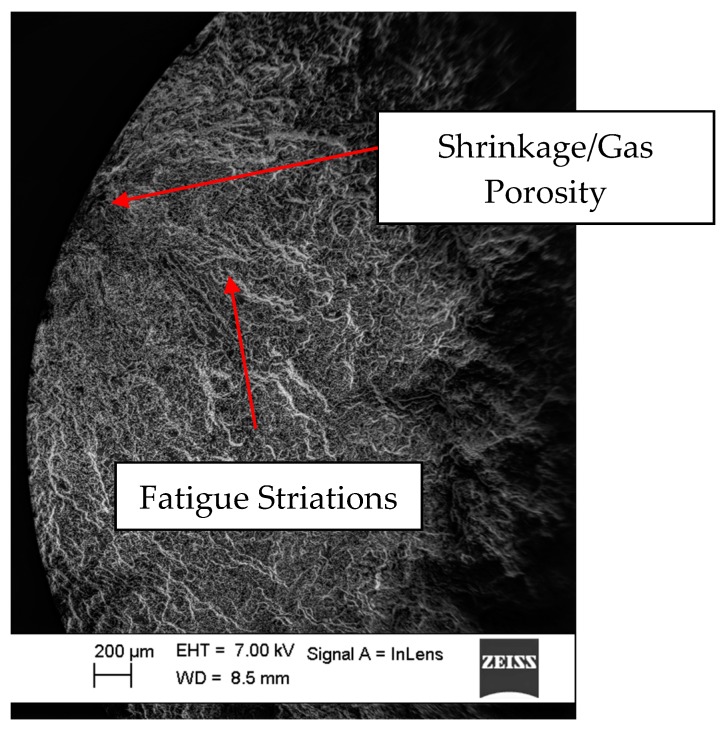
SEM micrograph showing the characteristic fatigue striations often observed in cyclic loading.

**Figure 21:**
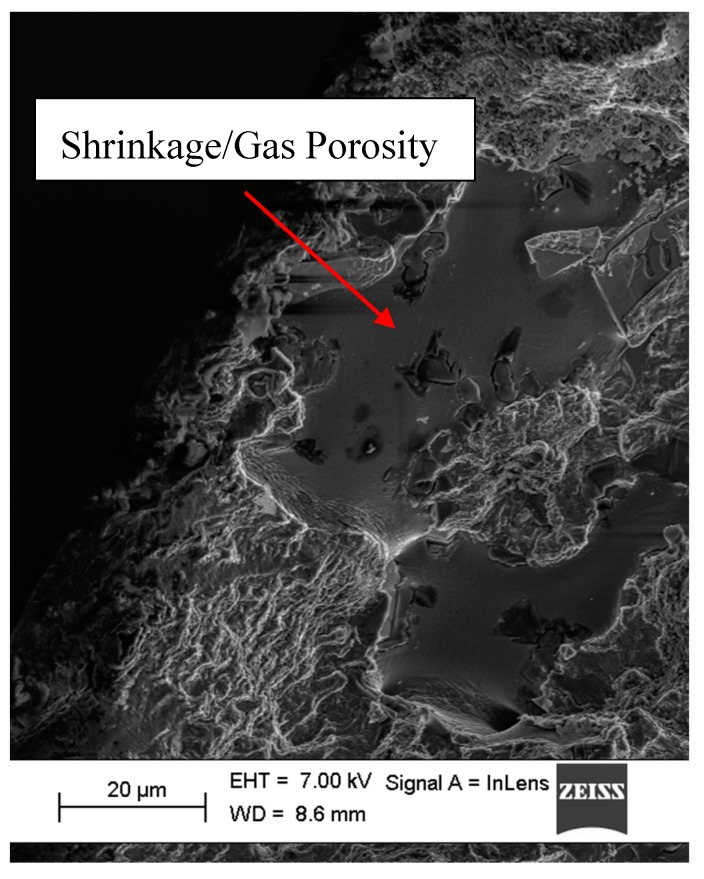
SEM micrograph indicating the crack origin location, a shrinkage or gas porosity.

The peak tensile stress response towards the end of the life of the tested samples during cyclic loading is oftentimes influenced by the location of the major crack developed in association with cyclic loading relative to the extensometer blades [21]. The stress peaks during the final stages of cyclic strain-controlled fatigue test close to the fracture could develop in one of two ways: 1. There is a decrease in peak stress development if the developed crack falls in the control volume between the mounted extensometer blades. 2. The stress peaks increase, however, if the cracks are located in the test volume outside the mounted extensometer blades. For the latter case, with the crack outside the gauge length of the extensometer, the machine applies excess loads when the crack has developed to a significant length to enforce the prescribed strain cycles in the volume between the extensometer blades resulting in a faster fracture. Except for one specimen (ε_amp_ 0.2%-S02), all the other samples had their cracks fall within the extensometer blades resulting in a reduced tensile stress development closer to failure as can be seen in Figure 6. Figure 20 and Figure 21 show a typical crack initiation point, from gas or shrinkage porosity, during the cyclic strain-controlled fatigue loading of the specimens. The controlled propagation of the cracks can be clearly observed in the form of fatigue striations before the eventual uncontrolled catastrophic failure.

### 4.3. Numerical Modelling

#### 4.3.1. Modelling of Monotonic Deformation Curves: Ramberg-Osgood Model

The monotonic deformation behaviour of the material can be modelled with a power law type equation that relates the plastic strain to the stress as proposed by Ramberg-Osgood [38]. The stress-strain relationship is an additive partition of the elastic and plastic components that add up to give the total strain in the material and is especially suitable for materials that do not exhibit a distinct yield point [15].

Ramberg Osgood Power Law:

(1) $${\sigma \;=\;H\varepsilon }^{P}$$

Tensile Stress-Strain Model:

(2) $$\varepsilon_{\mathrm{total}}{\;=\;\varepsilon }_{\mathrm{plastic}}{\;+\;\varepsilon }_{\mathrm{elastic}}$$

(3) $$\varepsilon_{\mathrm{total}}\;=\;\frac{\sigma }{E}\;+\;{\left(\frac{\sigma }{H}\right)}^{\frac{1}{n}}$$

**Figure 22:**
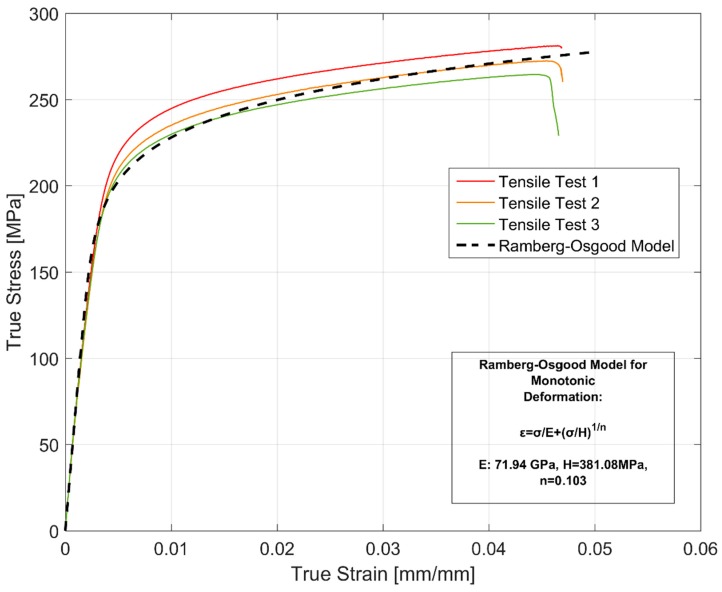
Ramberg-Osgood model for the material’s tensile stress-strain behaviour.

Where is the true stress, ε^P^ is the plastic strain, E is the material’s Young’s Modulus, H is a material constant and n is the strain hardening exponent. Since the strains are large in the tensile tests performed, the model parameters have been calibrated against the true stress—true strain data. A least square optimization routine was used to calibrate the model parameters against the three tensile test data and the results are as shown in Figure 22.

#### 4.3.2. Modelling of Cyclic Hardening Curves

Owing to the continuously varying nature of the peak stresses developed in cyclic loading due to cyclic hardening or softening, peak stresses obtained at half the life of such tests are used to develop the so called cyclic stress-strain curves [15]. However, the peak stresses associated with tensile and compressive loading are often asymmetric in their development as can be seen in Figure 6, and hence, their numerical mean is employed for developing the cyclic hardening curves representing the relationship between the stress and strain amplitudes for the cyclic loading of the tested A356-T7 alloy.

Ramberg-Osgood Model for Cyclic Hardening:

As there is no distinct yield point of the cyclic stress strain curve, as is often the case with cyclic hardening curves [15], a Ramberg-Osgood type equation is used to model the cyclic stress-strain curve similar to the monotonic stress-strain behaviour modelling. As the plastic strain amplitudes obtained at tests with 0.2% total strain amplitude and the equivalent stress-controlled tests are low, only the plastic strain amplitudes obtained at the higher loading levels of 0.3% and 0.4% total strain amplitudes and the corresponding stress-controlled test data were calibrated against the Ramberg-Osgood model described below:

Cyclic Stress-Strain Model:

(4) $${\text{ε}}_{\mathrm{amp}}=\frac{{\text{σ}}_{\mathrm{amp}}}{\text{E}}\;+(\frac{{\text{σ}}_{\mathrm{amp}}}{{\text{H}}^{\prime }})\frac{1}{{\text{n}}^{\prime }}$$

**Figure 23:**
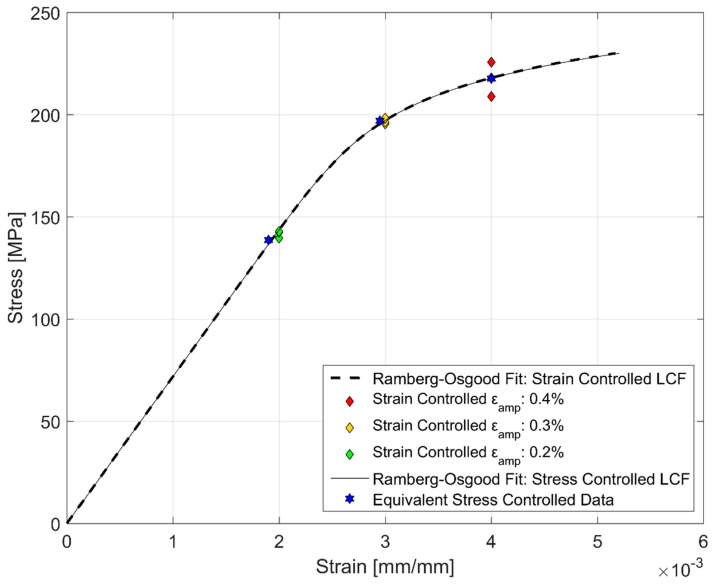
Cyclic hardening behaviour of A356-T7 from strain and stress-controlled fatigue tests.

Here σ_amp_, ε_amp_ being the stress and strain amplitudes respectively. H′ and the cyclic strain hardening exponent n′ are different from the monotonic curves and are determined using the least squares fitting of the test data to the model parameters described above and are presented in Table 7. The average peak stress and strain obtained at half the life in the strain and stress controlled fatigue tests and the corresponding Ramberg-Osgood cyclic hardening curves are presented in Figure 23. It can be seen from Figure 23 and Table 7 that the load-response curves are nearly identical regardless of the strain or stress that was used to control the cyclic deformation. The hardness curves developed using the stabilized stress and strain amplitudes obtained at half the life of the components is presented in Figure 23. Azadi et al. [17] studied the differences between the monotonic hardening behaviour of A356-T6 alloys between the stress and strain controlled loadings and observed no discernible differences. A similar indifference in the cyclic hardening behaviour is observed here for the over-aged A356-T7 alloys. 

#### 4.3.3. Constitutive Modelling

For efficient CAE procedures in the development of any component, reliable material models that link the state of stress and strain are often desired. A material model is a mathematical simplification that relates the load to the material response. While the use of multi-scale modelling approaches are enticing, they are often intricate and computationally expensive on an industrial context [39]. This study instead uses the other popular technique of macroscopic phenomenological approach that is a much faster and computationally inexpensive method to model the material plasticity. While a number of different physical behaviours could be modelled, one often chooses a degree of complexity that is adequately accurate without becoming inessentially complex and computationally expensive [40]. As can be seen in Figure 6, the shakedown of the hysteresis loops occurs quite fast and remain fairly stable until the fatigue failure for the completely reversed loads. While the material does exhibit a strong ratchetting and mean stress relaxation behaviour, the load asymmetry in a cylinder head is often not significant in real life and hence is not considered further for modelling.

The tensile and compressive stresses are not symmetric about the axis indicating the translation of the yield surface in reversed loading conditions necessitating the need for a kinematic hardening parameter in the model. The yield surface evolution in Figure 8 also indicates a constant increase in the yield surface and the elastic range with consecutive cyclic loads indicating the need to model the isotropic hardening part. A look at the hysteresis loops and the stress evolution in Figure 18 and Figure 19 indicate that the material exhibits the said hardening behaviour that saturates quite rapidly in the initial few cycles and remains fairly stable until failure necessitating a non-linear kinematic and isotropic combined hardening model [40].

To model the cyclic hardening behaviour of the A356-T7 aluminium alloys, a rate independent nonlinear isotropic-kinematic combined hardening model as implemented in a commercial software such as Abaqus [41] is used. The kinematic hardening is modelled using a linear Ziegler hardening law together with a recall/relaxation term that accounts for the non-linearity. To improve the model prediction, several kinematic hardening components can be superposed.

Yield Surface is defined by the following function:

(5) $$F\;=\;f\left(\sigma -\alpha \right){-\sigma }^{0}\;=\;0$$

Where α is the backstress (kinematic hardening component) and $\sigma^{0}$ is the equivalent stress defining the size of the yield surface. The linear Ziegler hardening law with multiple backstresses is defined as below [41]:

Kinematic Hardening Law:

(6) $${\dot{\alpha }}_{k}{=}_{C_{k}}\frac{1}{\sigma^{0}}\left(\sigma -\alpha \right){\dot{\bar{\varepsilon }}}^{\mathrm{pl}}{-\;\gamma }_{k}\alpha_{k}{\dot{\bar{\varepsilon }}}^{\mathrm{pl}}$$

Overall backstress:

(7) $$\alpha \;=\overset{N}{\underset{k=1}{\sum }}\alpha_{k}$$

Where C is the initial kinematic hardening moduli, γ determines the rate at which the kinematic hardening moduli decreases with increasing plastic deformation, $\sigma^{0}$ is the equivalent stress defining the size of the yield surface, α is the backstress and ${\dot{\bar{\varepsilon }}}^{\mathrm{pl}}$ is the is the equivalent plastic strain rate. The change in the size of the yield surface is modelled using an exponential law [41] as below:

Exponential Law:

(8) $$\sigma^{0}{\;=\;\sigma ¦}_{0}{\;+\;Q}_{\infty }\left(1-e^{-b{\bar{\varepsilon }}^{\mathrm{pl}}}\right)$$

Where $\sigma {¦}_{0}$ is the yield at zero plastic strain, $Q_{\infty }$ is the maximum change in the size of the yield surface and b is the rate at which the size of the yield surface changes as plastic straining develops [41]. $C_{k},\gamma_{k},\;\sigma {¦}_{0},Q_{\infty },\;b$ are the material parameters that are calibrated against cyclic test data. For modelling the cyclic deformation behaviour of A356-T7, two backstresses are used with a single linear and non-linear kinematic hardening component each. The inverse problem to identify the model parameters is carried out by minimizing the error between the simulated and the experimental results. A uniaxial strain controlled completely reversed load tests with ε_amp_ = 0.4% and ε_mean_ = 0% is used to calibrate the model parameters as it is important to capture the cyclic plastic behaviour at large strain amplitudes. The commercial finite element software Abaqus with the said combined non-linear kinematic-isotropic hardening model implemented is used to replicate the uniaxial test and a non-gradient Nelder-Mead simplex algorithm is used to optimize the model parameters by minimizing the sum of squares of the difference between the simulated and experimental data. The sum of squares type method to optimize the parameter set specified by π is constructed by having the stress response of the experimental ($\bar{\sigma }$) and simulated (σ_i_) data set at identical time intervals (equates to identical strain levels) t_i_, i = 1,2,3…N in the following format [40]:

(9) $$F\;(\pi )\;=\frac{1}{2}\;\sum_{i=1}^{N}[\sigma_{\text{i}}\left({\pi ,t}_{i}\right)-\bar{\sigma }(t_{1})]{}^{2}$$

**Figure 24:**
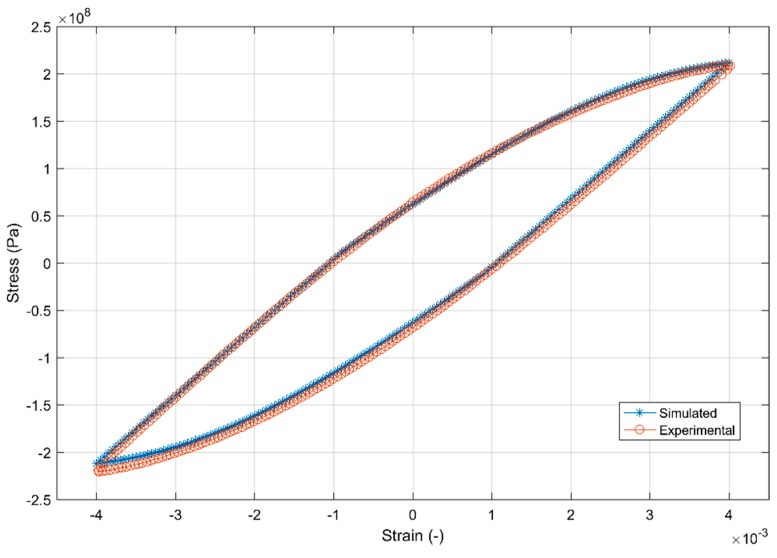
Model prediction versus experimental data of the 2nd strain cycle.

**Figure 25:**
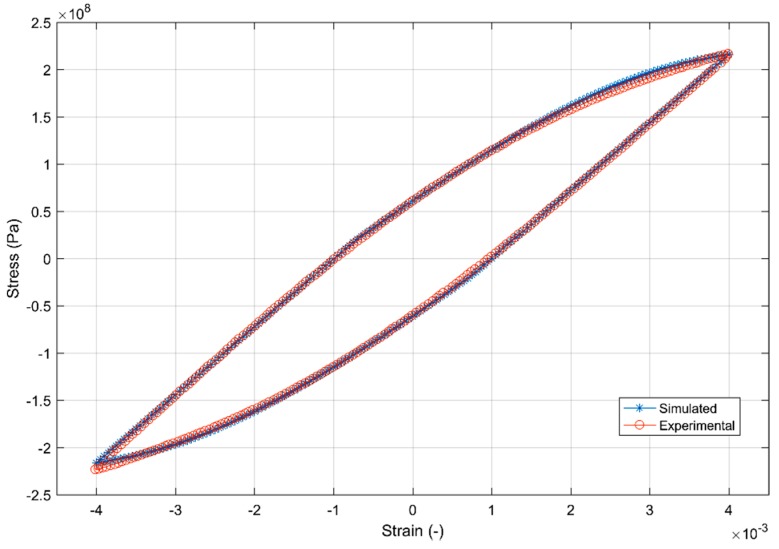
Model prediction versus experimental data of the 20th strain cycle.

The first 20 cycles of the simulated and experimental data set were used to calibrate the combined hardening model parameters and the summary of the calibrated models is as presented in Table 8. Figure 24 and Figure 25 show the fit of the model against the test data for the 2nd and the 20th strain load cycles.

#### 4.3.4. Fatigue Criterion

Once the load response history of the material subjected to the applied operational loads is determined, the key subsequent step is to estimate the life of the component using the obtained stress-strain history and a suitable failure criteria. While life determination could be coupled with the constitutive models, multiple studies have shown that despite involving more complicated model parameter calibration and increased computational cost, no improvement was observed in the lives predicted [29,42,43,44]. Hence, a decoupled constitutive behaviour and fatigue life modelling is often adopted in the industry. Considering the life of a cylinder head, during the operation cycle, the nature of the loads is often multi-axial and non-isothermal which leads to constant discussions over the suitability of different failure criteria that could be employed to estimate the life of structures. 

Parametric approaches, like the ones pioneered by Coffin [45] and Manson [46], are often used to model the life of the material in the automotive industry as a function of the applied strain amplitude at various temperatures and are quite practical while providing representative life estimates [39]. However, these models often need further modification to account for the mean strain and stress effects [15]. The Smith-Watson-Topper [47] and the popular Ostergren fatigue criterion are often used to account for such complex loading scenarios while estimating the fatigue life. 

**Figure 26:**
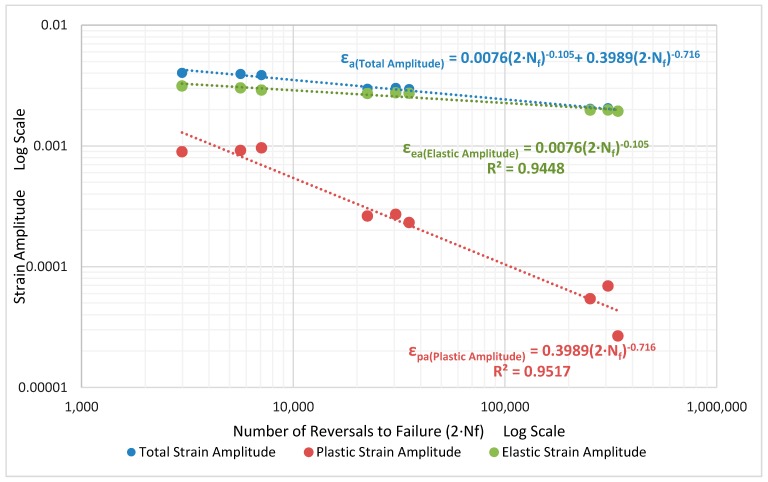
Coffin-Manson strain life plot.

The strain life curve obtained with the previously described completely reversed loads with total strain amplitudes of 0.2, 0.3 and 0.4% and the strain ratio of R_ε_ = −1 is presented in Figure 26. The total strain amplitude (ε_a_) is the additive partition of the elastic and plastic strain amplitudes as shown below:

ε_a_ = ε_ea_ + ε_pa_ (10)

Where, ε_ea_ is estimated using the stress amplitude σ_Amp_ and the young’s modulus E using the relation ε_ea_ = σ_Amp_/E. The plastic strain amplitude is taken as half the width of the hysteresis loop. All the parameters are estimated from the hysteresis loop at half the life (N_f_/2) of the specimens where stabilization is considered to have occurred [15]. By plotting the life (N_f_) or strain reversals (2∙N_f_) against the different strain amplitudes (ε_ea_ and ε_pa_), the parameters of the Coffin-Manson relationship can be obtained as detailed below:

Coffin-Manson Relationship:

ε_a_ = ε_ea_ + ε_pa_ (11)

(12) $$\varepsilon_{a}=\;\frac{\sigma_{f}^{\prime }}{E}{\left({2N}_{f}\right)}^{b}{\;+\;\varepsilon }_{f}^{\prime }\;{\left({2N}_{f}\right)}^{c}$$

Here, the elastic strain amplitude is represented by:

(13) $$\varepsilon_{\mathrm{ea}}\;=\;\frac{\sigma_{a}}{E}\;=\;\frac{\sigma_{f}^{\prime }}{E}{\left({2N}_{f}\right)}^{b}$$

The plastic strain amplitude by:

(14) $$\varepsilon_{\mathrm{pa}}{\;=\;\varepsilon }_{f}^{\prime }\;{\left({2N}_{f}\right)}^{c}\;$$

The estimated parameters for the strain-life curve are presented in Figure 26 and Table 9. From the strain-life curve in Figure 26, it can be seen that the plastic strain amplitude-life curve has a steeper slope compared to the elastic strain amplitude-life curve as with most metals [15]. The model has been estimated over a wide range of loads and lives, between 2800 and approximately 200,000 cycles, and hence can be used in situations that show both high and negligible plastic strains.

## 5. Conclusions

The A356-T7 + 0.5 wt.% Cu alloys extracted from cylinder heads were tested monotonically and cyclically to study the deformation behaviour of the alloy. The cyclic tests were conducted with equivalent stress and strain control to observe the equivalence of the different modes of control and its effect on the material response and fatigue lives.

- Under monotonic loading, the material yielded on average at 210MPa and exhibited a hardening behaviour with the peak tensile tress reaching values up to 272 MPa on average. There exists a scatter of some 5% in the yield and tensile strength observed between the replicas, while the fracture strains are consistent at 4.7% between the replicas tested.
- Under cyclic loading, the material exhibits slight cyclic hardening for all load levels with the stress amplitudes increasing by up to 7–9% from the initial cycle for all tested load levels. As with monotonic loading, the cyclic tests exhibit considerable scatter in the stress response between the replicas.
- While comparing the effect of different loading modes, namely, the cyclic strain and cyclic stress controlled tests on the deformation behaviour and fatigue lives, it was observed that both the material hardening and the cycles to failure, N_f_, are quite similar between equivalent stabilized stress and strain load levels implying the equivalence of the two tested modes for the evaluation of fatigue lives.
- The material exhibits mean stress relaxation with both the tensile and compressive mean strains under the non-completely reversed strain controlled cyclic loading. On the other hand, this study only observed the complimentary ratchetting behaviour with tensile mean stress for the relatively small mean stresses imposed.
- The monotonic and cyclic hardening curves could be modelled accurately with a power law type Ramberg-Osgood deformation models.
- The continuum deformation behaviour of the material can be modelled using a rate independent nonlinear isotropic-kinematic combined hardening model.

## Figures and Tables

**Table 1:** Chemical composition of A356-T7 as tested in wt%.

Si	Cu	Mg	Ti	Fe	Mn	Others	Al
6.8	0.53	0.35	0.12	0.10	0.07	<0.05	Bal

**Table 2:** Summary of mechanical tests.

Series	Nature of Test	Loading Condition	Number of Tests Run
1	Uniaxial strain controlled tensile tests	Strain rate: 1 × 10^−4^ s^−1^	3
2	Uniaxial, completely reversed total strain-controlled fatigue tests	Strain rate: 1 × 10^−2^ s^−1^, Strain amplitudes: 0.2, 0.3 & 0.4%	3 × 3
3	Uniaxial, non-completely reversed total strain-controlled fatigue tests	Strain rate: 1 × 10^−2^ s^−1^, Strain amplitudes: 0.4%, Mean strain: +0.2%	1
4	Uniaxial, non-completely reversed total strain-controlled fatigue tests	Strain rate: 1 × 10^−2^ s^−1^, Strain amplitudes: 0.4%, Mean strain: −0.2%	1
5	Uniaxial, equivalent stress-controlled fatigue tests	Strain rate: ca 1 × 10^−2^ s^−1^	4 × 1

**Table 3:** Mechanical properties of A356-T7 estimated from true stress versus true strain tensile test data.

Sample	Young’s Modulus (*E*) GPa	Yield Strength (*R_p_*_0.2_) MPa	Ultimate Tensile Strength (*R*_m_) MPa	Fracture Strain (*ε*_f_) %
1	73	204	265	4.7
2	71	219	281	4.7
3	75	207	272	4.7
Average	73	210	272	4.7

**Table 4:** Summary of stress development and the life of the strain-controlled fatigue samples.

Sample	True Stress Range (Δ*σ*) in 1st Cycle (MPa)	Peak True Stress Range Developed Before Failure (MPa)	% Increase in Peak True Stress Range Before Failure	Life (*N*_f_)
ε_amp_ 0.4%, S01	405	441	8.9	2818
ε_amp_ 0.4%, S02	390	421	7.9	3540
ε_amp_ 0.4%, S03	416	455	9.3	1492
ε_amp_ 0.3%, S01	364	395	8.6	11,204
ε_amp_ 0.3%, S02	367	399	8.7	17,636
ε_amp_ 0.3%, S03	368	400	8.6	15,238
ε_amp_ 0.2%, S01	262	285	8.6	170,752
ε_amp_ 0.2%, S02	269	290	7.7	126,308
ε_amp_ 0.2%, S03	271	292	7.4	153,248

**Table 5:** Stress data from strain-controlled fatigue tests used for equivalent stress-controlled tests.

Strain Controlled Test	Max Stress (Peak) At N_f_/2 (MPa)	Min Stress (Trough) At N_f_/2 (MPa)	Mean Stress At N_f_/2 (Mpa)	Life N_f_
ε_Amp_: 0.2%, R_ε_ = −1	137	−142	−2.76	170,752
ε_Amp_: 0.3%, R_ε_ = −1	195	−200	−2.54	17,636
ε_Amp_: 0.4%, R_ε_ = −1	214	−221	−3.54	2818
ε_Amp_: 0.4%, R_ε_ = −3*	221	−214	+3.25	624

* The stresses obtained at N_f_/2 for the asymmetric strain-controlled loading is the residual after the mean stress relaxation and differ significantly from the initial cycles unlike the other completely reversed strain controlled.

**Table 6:** Comparison of the life of specimens in strain and equivalent stress-controlled fatigue tests.

Strain Controlled Test	Life N_f_	Equivalent Stress Controlled Test (MPa)	Life N_f_
ε_Amp_: 0.2%, R_ε_ = −1	170,752	σ_Mean_: −2.76, σ_Amp_: 139.30	121,714
ε_Amp_: 0.3%, R_ε_ = −1	17,636	σ_Mean_: −2.54, σ_Amp_: 197.33	14,959
ε_Amp_: 0.4%, R_ε_ = −1	2,818	σ_Mean_: −3.54, σ_Amp_: 217.78	2,578
**ε_Amp_: 0.4%, R_ε_ = −3	**624	**σ_Mean_: +3.25, σ_Amp_: 217.37	**3,239

** The stresses obtained at N_f_/2 for the asymmetric strain-controlled loading is the residual after the mean stress relaxation and differ significantly from the initial cycles unlike the other completely reversed strain-controlled tests and hence, the lives can’t be directly compared. The objective of this test is instead to see the effect of a positive mean strain on ratchetting/shake down.

**Table 7:** Ramberg-Osgood model parameters for cyclic hardening.

Nature of Cyclic Test	Young’s Modulus (GPa)	Offset Yield Strength $\sigma_{0}^{\prime }$ (MPa)	H′ (MPa)	Cyclic Strain Hardening Coefficient n′
Strain controlled cyclic loading	72	230	368.79	0.0758
Stress controlled cyclic loading	72	230	366.68	0.0751

**Table 8:** Model parameters of the non-linear combined isotropic-kinematic hardening model.

Young’s Modulus (Pa)	Yield Stress at Zero Plastic Strain (Pa)	Kinematic Hardening Parameter C1 (Pa)	Gamma 1 (-)	Kinematic Hardening Parameter C2 (Pa)	Gamma 2 (-)	Q-Infinity (Pa)	Hardening Parameter b (-)
71.94 × 10^9^	1.108 × 10^8^	1.708 × 10^11^	1616	1.518 × 10^9^	0	55 × 10^6^	1.5

**Table 9:** Coffin-Manson model parameters for the strain-life curve.

Parameter	E [GPa]	σ_f_′ [MPa]	b	ε_f_′	c
Estimation	72	547	−0.105	0.3989	−0.716

## Data statement

The raw/processed data required to reproduce these findings cannot be shared at this time as the data also forms part of an ongoing study.
